# Entropy Effects in Intermolecular Associations of Crown-Ethers and Cyclodextrins with Amino Acids in Aqueous and in Non-Aqueous Media

**DOI:** 10.3390/e24010024

**Published:** 2021-12-24

**Authors:** Tatyana Usacheva, Irina Terekhova, Diana Alister, Mikhail Agafonov, Natalya Kuranova, Dmitry Tyurin, Valentin Sharnin

**Affiliations:** 1 Department of Inorganic Chemistry and Technology, Ivanovo State University of Chemistry and Technology, 153000 Ivanovo, Russia; matchoaa@mail.ru (D.A.); kuranova_nn@isuct.ru (N.K.); sharn@isuct.ru (V.S.); 2 G.A. Krestov Institute of Solution Chemistry of the Russian Academy of Sciences, 153045 Ivanovo, Russia; ivt@isc-ras.ru (I.T.); ama@isc-ras.ru (M.A.); 3 Department of Organic Chemistry and Technology, Ivanovo State University of Chemistry and Technology, 153000 Ivanovo, Russia; dimitar1991@yandex.ru

**Keywords:** amino acids, 18-crown-6, cyclodextrins, complex formation, thermodynamics, compensation effect

## Abstract

The analysis of the ratios of entropy and enthalpy characteristics and their contributions to the change in the Gibbs energy of intermolecular interactions of crown ethers and cyclodextrins with amino acids is carried out. Two different types of macrocycles were chosen for examination: crown ethers with a hydrophilic interior and cyclodextrins with a hydrophobic inner cavity and a hydrophilic exterior. The thermodynamics of complex formation of crown ethers and cyclodextrins with amino acids in water and aqueous-organic solvents of variable composition was examined. The contributions of the entropy solvation of complexes of 18-crown-6 with glycine, alanine, phenylalanine to the change in the entropy of complexation in water-ethanol and water-dimethyl sulfoxide solvents was calculated and analyzed. It was found that the ratios of the entropy and enthalpy solvation of the reagents for these systems have similar trends when moving from water to aqueous-organic mixtures. The relationship between the thermodynamic characteristics and structural features of the complexation processes between cyclodextrins and amino acids has been established. The thermodynamic enthalpy–entropy compensation effect was revealed, and its features for complexation of cyclodextrins and 18-crown-6 were considered. It was concluded that, based on the thermodynamic parameters of molecular complexation, one could judge the mode of the formation of complexes, the main driving forces of the interactions, and the degree of desolvation.

## 1. Introduction

The study of the “guest–host” molecular recognition phenomenon began with the works of J.-M. Len, C. Pedersen, and D. Kram, who received the Nobel Prize in Chemistry in 1987 for the development and application of molecules with structurally specific interactions with high selectivity [1,2,3,4]. Currently, fundamental and applied sciences are still interested in studying the unique selective properties of macrocycles [5,6,7,8].

Assessment and prediction of stability constants of complexes depending on the structure of guest and host molecules, as well as the nature and composition of the solvent, analysis of structural characteristics and thermodynamic parameters of formation of molecular complexes of macrocycles are relevant both from the point of view of the development of supramolecular chemistry, and priority fields of biochemistry, biotechnology, and pharmacology.

Unlike such thermodynamic parameters of complexation and solvation of reagents as the Gibbs energy change and the enthalpy change, which can be obtained by experimental methods, the entropy change of these processes can be only calculated on the basis of experimentally obtained values of Δ*G* and Δ*H*.

Usually, less attention is paid to the analysis of entropy than to the analysis of enthalpy characteristics of reactions and solvation of reagents. Entropy is often considered as a measure of the disorder of the system, related to the number of free particles in the system and reflecting the contribution of changes caused by the reorganization of the solvent and/or solvate shells of particles in solution. In this regard, it is logical to assume that the growth of entropy can characterize the processes of solvation of reagents, dissociation of complexes, and the release of solvent molecules from the solvation shells of reagents during the complexation.

In this paper, the enthalpy–entropy compensation effect in “guest–host” molecular complexation of crown ethers and cyclodextrins as host molecules with amino acids as guests is analyzed. Crown ethers are macroheterocyclic compounds containing more than 11 atoms in their cycles, of which at least four are heteroatoms that are interconnected by ethylene bridges. Usually, an oxygen atom is a heteroatom.

Cyclodextrins (CDs,) are an independent class of macrocyclic compounds obtained during the enzymatic disintegration of starch and consisting of 6-8 glucose units closed in a cycle. Unlike crown ethers, these compounds are of natural origin and, due to this, they are widely used in pharmaceuticals, biotechnology, cosmetology, and the food industry [9,10,11,12]. The second difference between CDs and crown ethers is the polarity of the macrocyclic cavity. In CDs, it is hydrophobic, while the outer surface of the molecules formed by hydroxyl groups is hydrophilic. In accordance with the principles of geometric and energy complementarity, lipophilic compounds can be located in the CD cavity and retained in it due to non-covalent interactions. The distinguished feature of the resulting inclusion complexes is their good solubility in aqueous media, and this determines the use of CD as solubilizers [13,14]. Thus, the main property of CDs, which determines the main directions of their practical use, is the ability to form inclusion complexes. In this regard, the ability of cyclodextrins to the inclusion complex formation is intensively studied, and the knowledge about the coordination of guest molecules in the host cavity, as well as about the stability of the resulting supramolecular complexes, has scientific and practical significance.

To examine the effects of enthalpy–entropy compensation in the intermolecular interactions, obtained thermodynamic parameters of complexation of 18-crown-6 (18C6) and cyclodextrins with amino acids (AA) in water and in water-organic mixed solvents were used [15,16,17,18,19,20,21,22,23,24,25]. The analysis of the solvation contributions of reagents to the change in the entropy of reactions of the formation of molecular complexes of 18-crown-6 with amino acids in water-organic mixed solvents was carried out using the literature data [15,16,17,18,19,20,21,22,26,27,28,29,30,31,32,33,34,35,36,37].

## 2. Results and Discussion

The formation of molecular complex between AA (guest) and macrocycle (host) in solution is described by the following equation:

guest_solv_ + host_solv_ ⇆ [guest ⊂ host]_solv_ (1)

The change in the free energy of complex formation, as well as the stability of the complexes is determined by the enthalpy and entropy contributions in accordance with the Gibbs–Helmholtz equation:

(2) $$\Delta_{c}G^{0}=\Delta_{c}H^{0}-T\Delta_{c}S^{0}$$

If the energy of the formation of a complex is determined by the Δ_c_*H*^0^ value, then the Δ_c_*S*^0^ value reflects all structural changes in the system.

It has been observed that complexation reactions involving biologically active molecules and simulating the processes occurring in the living organisms (for example, enzyme–substrate, peptide–DNA, antigen–antibody interactions, etc.) are accompanied by small negative changes of the free energy, despite the fact that changes in the enthalpy may be significant [38,39,40,41]. This can be explained by the phenomenon of enthalpy–entropy compensation, according to which, a favorable change in the enthalpy is often compensated by a parallel unfavorable (negative) change in the entropy, as a result of which Δ_c_*G*^0^ becomes insignificant. The linear relationship between Δ_c_*H*^0^ and Δ_c_*S*^0^ is mathematically expressed as follows:

(3) $$\Delta_{c}H^{0}=a+b\cdot \Delta_{c}S^{0}$$

where the coefficient *a* is a certain energy parameter that is practically not discussed in the literature [42], *b* is a compensation temperature. Equation (3) belongs to the category of “extrathermodynamic”, since it is an approximate and statistical relationship between the thermodynamic quantities and is not based on the thermodynamic laws. At *b* > 0, the enthalpy–entropy compensation occurs, and, conversely, at *b* < 0 an anti-compensation is observed [43].

To date, the nature of the enthalpy–entropy compensation effect attracts the attention of scientists, and there is no unified theory that could explain this phenomenon. There are two main points of view regarding the compensation effect in the literature. According to one of them, compensation effect is caused by the changes in the environment that are initiated by the course of reactions [44,45,46]. However, some authors argue that the compensation effect is an artifact and the observed linear correlation has no physical meaning, since it appears after transferring errors in the Δ_c_*H*^0^ and Δ_c_*S*^0^ calculation (especially applies to the use of the Van’t-Hoff method) [47]. However, to date, there have been enough publications that show the existence of a Δ_c_*H*^0^ = *f*(Δ_c_*S*^0^) correlation in the case of an independent determination of Δ_c_*H*^0^ and Δ_c_*S*^0^ [48,49].

The linear relationship between the changes of the enthalpy and entropy of complex formation is described as follows [48,50]:

(4) $$T\Delta \Delta_{c}S^{0}=\alpha \cdot \Delta \Delta_{c}H^{0}$$

When substituting the Equation (4) into the differential form of the Gibbs–Helmholtz equation

(5) $$\Delta_{c}G^{0}=\Delta \Delta_{c}H^{0}-T\Delta \Delta_{c}S^{0}$$

One can get the following

(6) $$\Delta_{c}G^{0}=(1-\alpha )\Delta \Delta_{c}H^{0}$$

Integration of Equation (4) leads to

(7) $$T\Delta_{c}S^{0}=\alpha \cdot \Delta_{c}H^{0}+T\Delta_{c}S_{0}^{0}$$

In Equations (4), (6) and (7), the coefficient *α* in its physical meaning is the degree of compensation. In other words, only (1 − *α*) of the enthalpy contribution goes to the direct stabilization of the complex, and the remaining *α* is fully compensated by the entropy contribution. The *T*Δ_c_*S*_0_^0^ value represents the intrinsic stability of the complex, i.e. the possibility of complex formation at Δ_c_*H*^0^ = 0 and Δ_c_*S*^0^ > 0. Very often *α* and *T*Δ_c_*S*_0_^0^ are interpreted as a measure of conformational changes of the macrocycle and desolvation of interacting particles, respectively [51,52].

Many authors associate the manifestation of the compensation effect with the reorganization of the solvent through the rearrangement of hydrogen bonds [49,53,54,55]. The idea is that the thermodynamic parameters of complexation are divided into two components—nominal and environmental [53,56]. The nominal part is associated with the interaction of solvated particles and the formation of a solvated complex, while the external part refers to the participation of solvent molecules in solvation/desolvation processes. Solvent molecules do not affect the value of the stability constant of the complex, i.e. the change in the free energy of the reorganization of the solvent is zero (Δ*G*_env_ = 0). Therefore, the correlation between Δ*H*_env_ and Δ*S*_env_ is the basis of the compensation effect.

From the standpoint of the solvation approach, the process of complexation in a simplified form can be represented as the transfer of molecules of a compound *A* from their own aqueous environment inside the solvent to the binding center of the ligand *B*, which is also surrounded by water molecules:

(8) $$A-w_{1}+B-w_{2}=A\cdot B+(w_{1}+w_{2})$$

As a result of this transfer, a complex *A·s* is formed, and the water molecules *w*_1_ and *w_2_* are released into a volumetric solvent. In this case, the Δ_c_*H*^0^ value will be determined by the energy balance of the H-bonds formed by the participants of the process before and after their interaction. On the other hand, the release of water molecules controlled by the entropy factor is accompanied by a redistribution of H-bonds. As we can see, the values of Δ_c_*H*^0^ and Δ_c_*S*^0^ are determined by the same process, which is associated with the formation and rearrangement of H-bonds. Thus, any strong intermolecular interaction (enthalpy factor) is compensated by a significant ordering of the system (entropy factor), resulting in a small Δ_c_*G*^0^ value.

The thermodynamic enthalpy-entropy compensation effect is widely considered in supramolecular chemistry for the processes of complexation of macrocyclic ligands [48,51]. When discussing *T*Δ_c_*S*^0^ = *f*(Δ_c_*H*^0^) dependences, the slope (α) and the intercept (*T*Δ_c_*S*_0_^0^) are mainly considered, which, respectively, serve as a measure of conformational changes and desolvation.

### 2.1. Thermodynamic Enthalpy–Entropy Compensation Effect in the Complexation of Cyclodextrins with Amino Acids in Water

**Figure 1:**
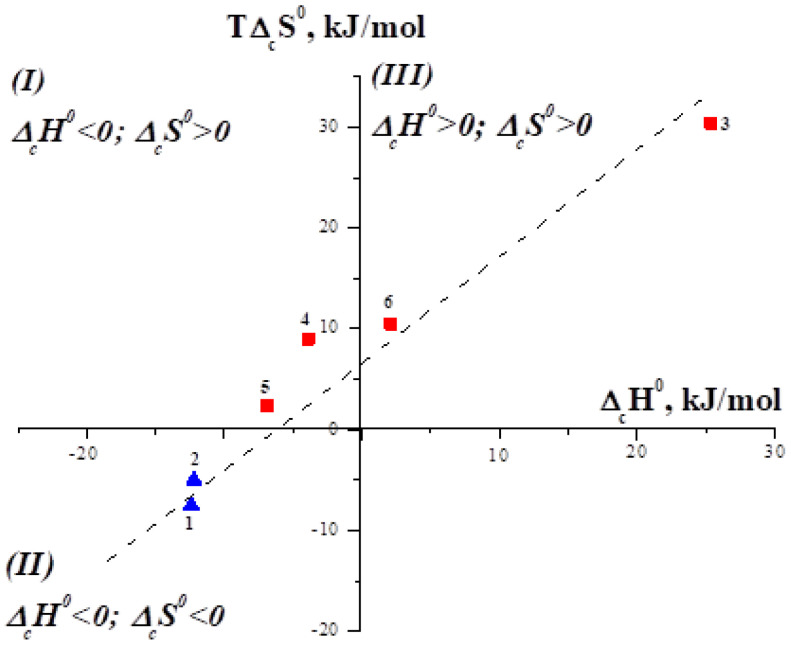
Dependence *T*Δ_c_*S*^0^ versus Δ_c_*H*^0^ for complex formation of cyclodextrins with aromatic amino acids in water at 298.15 K (1 [Phe⊂α-CD], 2 [Trp⊂α-CD], 3 [Phe⊂β-CD], 4 [Tyr⊂β-CD], 5 [Trp⊂β-CD], 6 [His⊂β-CD].

It is interesting to consider the compensation effect in the complexation reactions of α- and β-cyclodextrins with amino acids. It should be noted that CDs form 1:1 molecular complexes only with aromatic amino acids (Phe, Tyr, Trp and His) [25]. Figure 1 shows the dependence plotted for complexation of α- and β-cyclodextrins with amino acids in water [23,24]. As you can see, the dependence has a certain spread of points, which is determined by the fact that Δ_c_*G*^0^≠ const and lies within some limits.

The dependence shown in Figure 1 has a slope α equal to 0.94. As it was mentioned above and follows from Equations (4) and (6), only (1 − α) part of the Δ_c_*H*^0^ value is included in the Δ_c_*G*^0^ value, and the remaining α is fully compensated by Δ_c_*S*^0^. In other words, for the complexation of CDs with aromatic amino acids, 94% of the enthalpy gain is compensated by the entropy factor, and the remaining 6% go to the stabilization of the inclusion complexes.

Figure 1 shows three regions corresponding to enthalpy–entropy stabilized complexes (I), only enthalpy stabilized complexes (II), and only entropy (III) stabilized complexes. As one can see, complexes formed by α-CD belong to the second thermodynamic group, which is characterized by the negative enthalpy and entropy changes of complex formation. Complexes of amino acids with β-CD belong to the first and third groups, which are characterized by the positive changes of the entropy of complex formation. This phenomenon is not random and determined by the different driving forces of the interaction of host molecules with the same guest molecules and by the structural features of the macrocycles. α-CD and β-CD differ from each other in the size of the macrocyclic cavity. α-CD, consisting of six glucose residues, has the smallest molecular cavity, which can include small-sized guest molecules. Inclusion of larger guests is also possible, but it would be shallow. In this case, the surface interactions of the remaining part of the guest molecule with the polar outer surface of the α-CD are not excluded. As a rule, van der Waals interactions and the H-bonding between the polar groups of the guest and −OH groups of the host determine the exothermicity of α-CD binding and the increase in the structuring of the system. On the contrary, β-CD formed by seven glucopyranose units has a large cavity, that promotes the deeper inclusion of guest molecules. Complex formation of β-CD is accompanied by the release of water molecules not only from the solvation shells of the host and guest molecules, but also from the macrocyclic cavity. In this case, more water molecules are released into the bulk solvent resulting in the increase in the disorder of the system. Thus, dehydration makes a considerable contribution to the thermodynamics of the complexation of β-CD with aromatic amino acids.

The significant effect of the solvent on the complexation of β-cyclodextrins can be clearly seen from the expression written to the change of the entropy of the complexation:

(9) $$\Delta_{c}S=\Delta_{\mathrm{ass}}S+\Delta_{\mathrm{reorg}}S$$

where Δ_ass_*S* is the change in entropy due to a decrease in the number of particles under complexation; Δ_reorg_*S* is the change in the entropy caused by the rearrangement of water molecules during the dehydration/hydration of the particles. The value of Δ_ass_*S* should be the same for the complex formation of α- and β-cyclodextrins, while Δ_reorg_*S* should be slightly higher for the complexation of β-cyclodextrins, since dehydration in this case is more significant. The fact that Δ_reorg_*S* (β-CD) > Δ_reorg_*S* (α-CD) and, consequently, Δ_c_*S*(β-CD) > Δ_c_*S*(α-CD) is confirmed by the experimental data. For the complexation of α-CD and β-CD with the same guests, a more negative Δ_c_*S*^0^ was obtained in the case of α-CD binding.

### 2.2. Thermodynamic Enthalpy–Entropy Compensation Effect in the Complexation of Crown Ethers in Water and in Water-Organic Solvents

The linear enthalpy–entropy correlation for the reactions of the formation of molecular complexes [Gly⊂18C6], [Ala⊂18C6] and [Phe⊂18C6] in H_2_O-EtOH, H_2_O-DMSO, and H_2_O-Me_2_CO solvents is observed [57]. The dependences *T*Δ_c_*S*^0^ = *α*Δ_c_*H*^0^ + *T*Δ*S*_o_ are approximated by the least square method with slope 0.62 (H_2_O-EtOH), 0.80 (H_2_O-DMSO), 0.70 (H_2_O-Me_2_CO), which do not depend on the structure of guest molecule. The values of confidence in the approximation of these dependencies are 0.950, 0.944 and 0.969, respectively.

In the review [51], when analyzing an array of thermodynamic data on ion complexation involving macrocycles (crown ethers, cyclodextrins, macrocyclic antibiotics) and their acyclic analogues (glymes, podands) in water, water–alcohol and individual non-aqueous solvents, it was found that the parameter *α* in the Equation (7) is individual for each type of ligand and does not depend on the stoichiometry of the complexes formed, and the values of *T*Δ*S*_o_ positive and increasing in the series of glymes/podands, crown ethers, cryptands, antibiotics. The contribution of *T*Δ*S*_o_ reflects the increment of its own entropy during complexation, and is mainly due to the desolvation of the complexing cation. For ionic complexes formed by crown ethers, the value *α* = 0.76, which is consistent with the values of α calculated by us for molecular complexes of Gly, Ala and Phe with 18C6. The values of α < 1 confirm the enthalpy stabilization of the complexes.

For the reactions of the formation of molecular complexes of Gly, Ala and Phe with 18C6 in the aqueous-organic solvents under consideration, the contribution from the change in the solvent composition to the enthalpy-entropy correlation is obvious: in all cases, the less negative values of Δ_c_*H*^0^ and *T*Δ_c_*S*^0^ correspond to solvents with a high water content, and the more negative values are observed in solvents with a high content of a non-aqueous component. However, the enthalpy-entropy correlations reflect the total effect of the solvent’s influence on both changes in the solvate state of the reagents and changes in the thermodynamics of complex formation, which does not allow us to establish a key solvation contribution into complex formation. For this purpose, the effect of the solvent on the change in the solvate state of the reagents was analyzed and the subsequent analysis of the ratios of the solvation contributions of the reagents to the change in the thermodynamic parameters of reactions during the transition from water to water-organic mixtures was carried out.

### 2.3. Entropy Characteristics of Reagents and Reactions of Formation of Complexes of Amino Acids and Peptides with 18-Crown-6 in Aqueous Organic Solvents

Selective binding of molecules occurs due to their mutual complementarity. When conducting molecular complexation reactions in non-aqueous and water-organic solvents, in addition to the complementarity principle, it is also necessary to take into account the changes in the solvation state of the participants, since the thermodynamic characteristics of the reaction depend on the solvation state of the reagents. To do this, it is necessary to understand the role of the solvent not only as a medium, but also as a participant of the chemical interactions.

A wide number of experimental evidences has been accumulated, showing the extremely important role of the solvent as a means of controlling the chemical process [58,59,60]. In accordance with the solvation-thermodynamic approach the change in the thermodynamic characteristic of the reaction in a non-aqueous solvent (compared to an aqueous one) is the result of solvation contributions of complex [AA⊂18C6], 18C6 and AA molecules:

Δ_tr_*Y^0^*_c_ = Δ_c_*Y^0^*_S_ − Δ_c_*Y^0^*_W_ = Δ_tr_*Y^0^* _[AA__⊂ 18C6]_ − Δ_tr_*Y^0^*_18C6_ − Δ_tr_*Y^0^*_AA_ (10)

where Δ*Y*^0^ = Δ*G*^0^, Δ*H*^0^, Δ*S*^0^

This relationship is the basis of the solvation approach to the description of the role of the solvent in complexation reactions.

The Equation (11) can be applied to analyze the ratios between the entropy characteristics of [AA⊂18C6] complex formation reactions and solvation of the reagents:

*T*Δ_tr_*S^0^*_c_ = *T*Δ_tr_*S^0^*_[AA__⊂__18C6]_ − *T*Δ_tr_*S^0^*_18C6_ − *T*Δ_tr_*S^0^*_AA_ (11)

The *T*Δ_tr_*S^0^* characteristics of [AA⊂18C6] complex formations as well as of the solvation of 18C6 and AA were calculated from the following equation by using the Δ*G^0^* and Δ*H^0^* values obtained from experiments:

Δ_tr_*G^0^* = Δ_tr_*H^0^ − T*Δ_tr_*S^0^* (12)

In particularly, the Δ_tr_*G^0^* and Δ_tr_*H^0^* for the reactions were from [15,16,17,18,19,20,21,22]. The Δ_tr_*H^0^* and Δ_tr_*G^0^* values for 18C6 solvation were obtained in [26,27,28,29,30,31]. The solvation parameters for amino acids and peptides were from [32,33,34,35,36,37].

In the majority of publications that use the solvation approach to analyze the results obtained, the entropic term of the Gibbs energy is considered in comparison with the enthalpy one. Then, based on this, a conclusion is made about which of the components prevails over the other in the Gibbs energy change, i.e., what proportion of the total chemical energy of the system is associated with a change in enthalpy, and which is associated with a change in its structure.

Here, the analysis of the entropy characteristics of reagents and reactions is proposed to be applied to understand the reasons for the differences in key solvation factors in the change in the stability of molecular complexes formed by 18-crown-6 ether with amino acids and peptides, and the energy of reactions of their formation.

**Figure 2:**
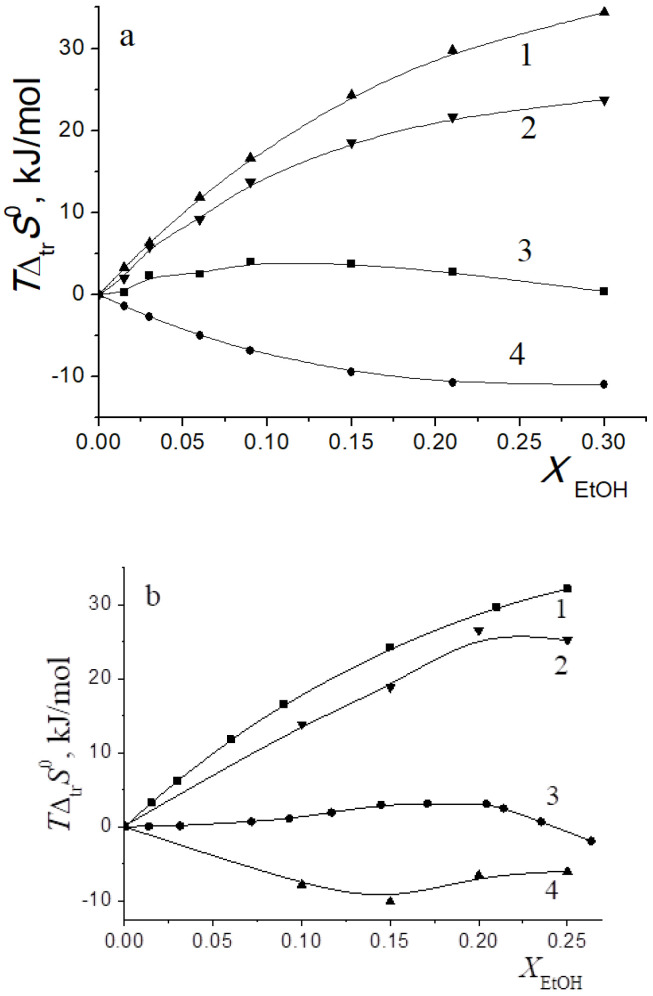
Entropy characteristics of complex formation reactions of [Gly⊂18C6] (**a**) and [3Gly⊂18C6] (**b**) in H_2_O-EtOH solvent. 1 *T*Δ_tr_*S*^0^(18C6); 2 *T*Δ_tr_*S*^0^([Gly⊂18C6]) (**a**), *T*Δ_tr_*S*^0^([3Gly⊂18C6]) (**b**); 3 *T*Δ_tr_*S*^0^(Gly) (**a**), *T*Δ_tr_*S*^0^(3Gly) (**b**); 4 *T*Δ_tr_*S*^0^_c_.

**Figure 3:**
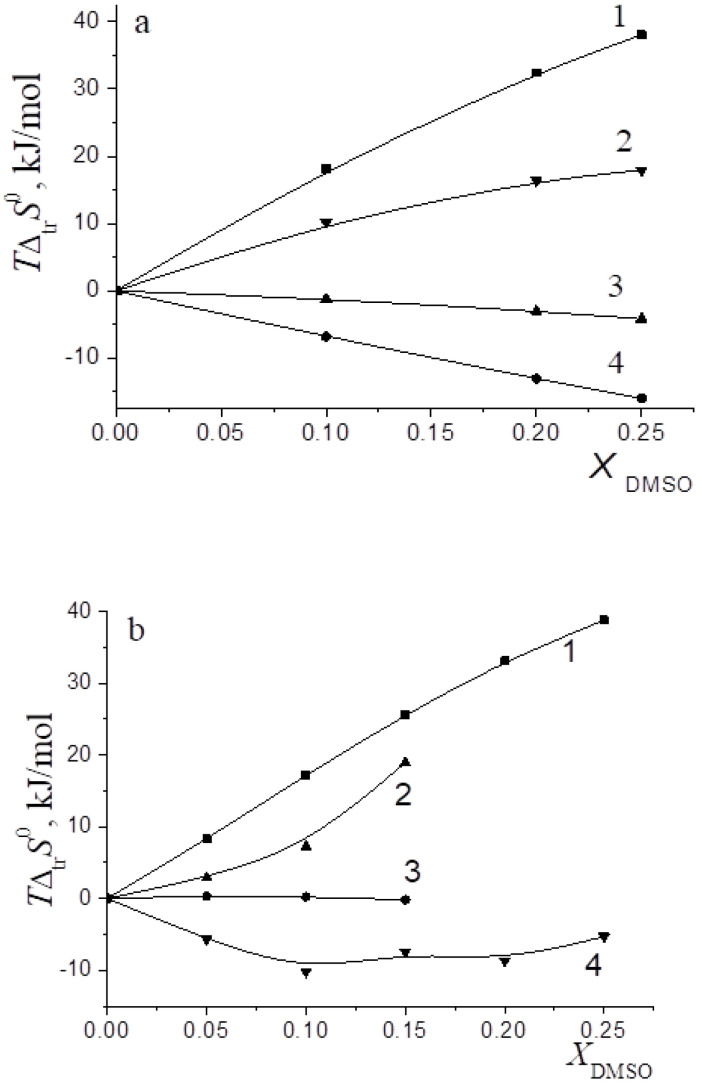
Entropy characteristics of complex formation reactions of [Gly⊂18C6] (**a**) and [3Gly⊂18C6] (**b**) in H_2_O-DMSO solvent. 1 *T*Δ_tr_*S*^0^(18C6); 2 *T*Δ_tr_*S*^0^([Gly⊂18C6]); 3 *T*Δ_tr_*S*^0^(Gly); 4 *T*Δ_tr_*S*^0^_c_.

**Figure 4:**
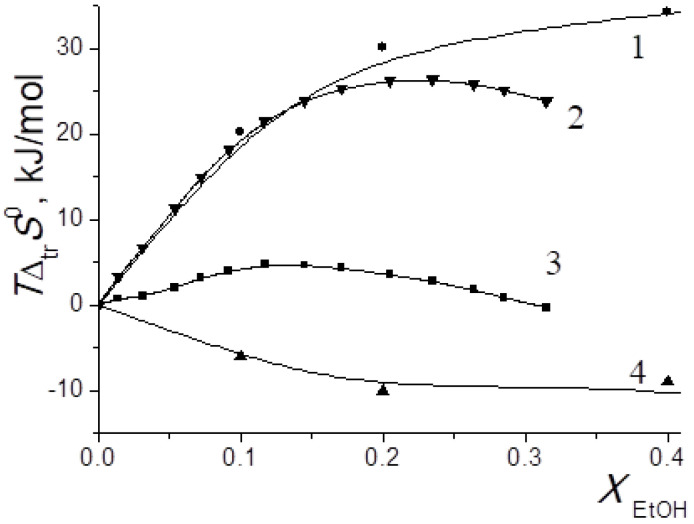
Entropy characteristics of complex formation reaction of [Ala⊂18C6] in H_2_O-EtOH solvent. 1 *T*Δ_tr_*S*^0^(18C6); 2 *T*Δ_tr_*S*^0^([Ala⊂18C6]); 3 *T*Δ_tr_*S*^0^(Ala); 4 *T*Δ_tr_*S*^0^_c_.

**Figure 5:**
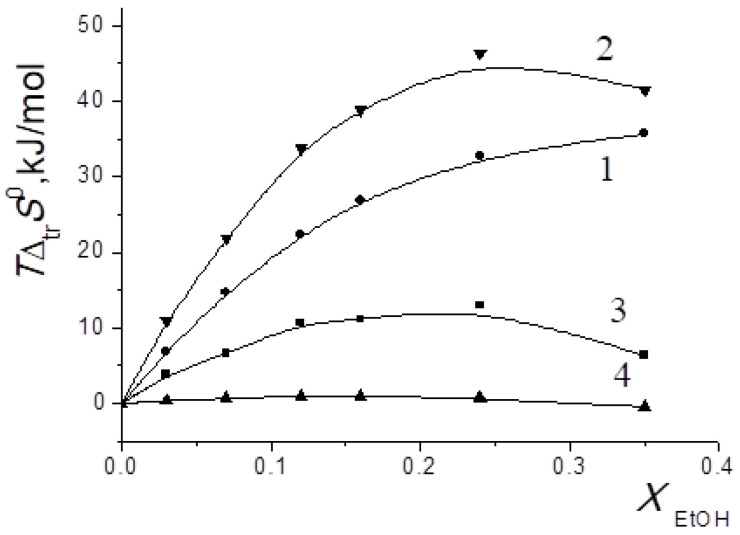
Entropy characteristics of complex formation reaction of [Phe⊂18C6] in H_2_O-EtOH. 1 *T*Δ_tr_*S*^0^(18C6); 2 *T*Δ_tr_*S*^0^([Phe⊂18C6]); 3 *T*Δ_tr_*S*^0^(Phe); 4 *T*Δ_tr_*S*^0^_c_.

In [15,16,17,18,19,20,21,22,29,57,59,61,62,63,64,65], we found that the transition from water to water-organic solvents leads to an increase in the stability of molecular complexes of 18-crown-6 with glycine, D,L-alanine, L-phenylalanine and glycyl-glycyl-glycine and to an increase in the exothermicity of reactions of their formation. It was shown that the contribution from the weakening of the solvation of the “guest” determines the growth of stability of molecular complexes during the transition from water to water-organic mixtures. In turn, the enthalpy of complex formation is mainly controlled by changes in the enthalpy of macrocycle solvation. This allows to predict the changes in the stability of molecular complexes by changing the solvation of “guest” molecules, and to estimate changes in the enthalpy of complex formation by changing the enthalpy of macrocycle solvation. The ratios of entropy characteristics of solvation of reagents and complexation reactions are similar to the ratios of the corresponding enthalpy parameters: for aliphatic amino acids and peptides the following ratios are observed *T*Δ_tr_*S*^0^ (AA) < *T*Δ_tr_*S*^0^ ([AA⊂18C6]) < *T*Δ_tr_*S*^0^ (18C6), and for aromatic AA the ratio is *T*Δ_tr_*S*^0^ (AA) < *T*Δ_tr_*S*^0^ (18C6) < *T*Δ_tr_*S*^0^ ([AA⊂18C6]) (Figure 2, Figure 3, Figure 4 and Figure 5).

The analysis of entropic solvation contributions to the Gibbs energy change for the formation of molecular complexes [Gly⊂18C6] in H_2_O-DMSO solvent (Figure 3) and [Ala⊂18C6] in H_2_O-EtOH solvent (Figure 4) can be explained by the limitation of available literature data on changes in the thermodynamic parameters of amino acid solvation in the corresponding solvents [34,35,37].

**Figure 6:**
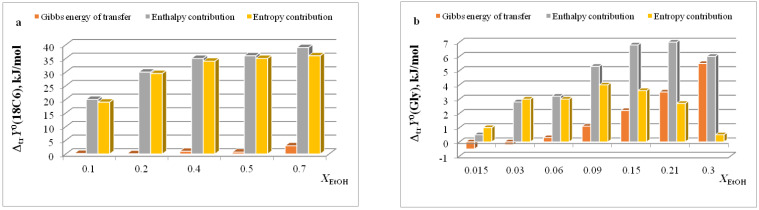
Ratios of thermodynamic parameters of transfer of 18C6 (**a**) and Gly (**b**) in H_2_O-EtOH.

**Figure 7:**
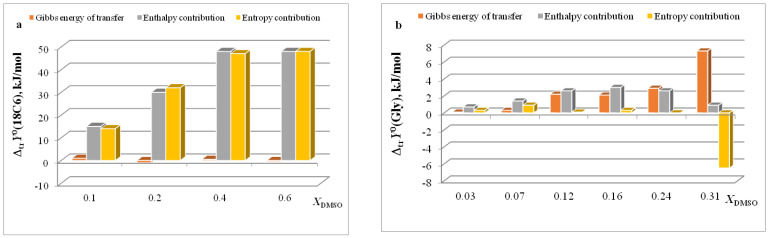
Ratios of thermodynamic parameters of transfer of 18C6 (**a**) and Gly (**b**) in H_2_O-DMSO.

**Figure 8:**
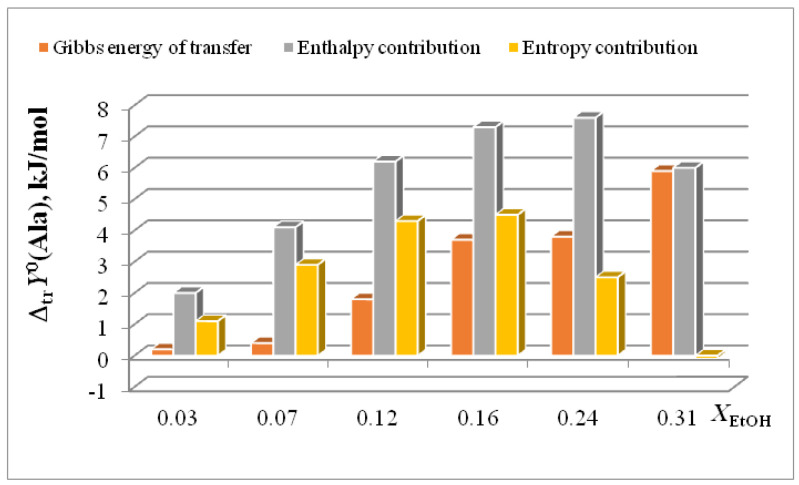
Ratios of thermodynamic parameters of transfer of Ala in H_2_O-EtOH.

**Figure 9:**
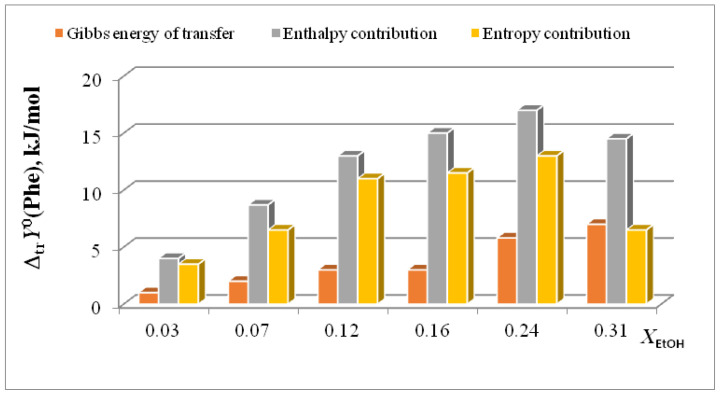
Relations of thermodynamic parameters of transfer of Phe in H_2_O–EtOH.

**Figure 10:**
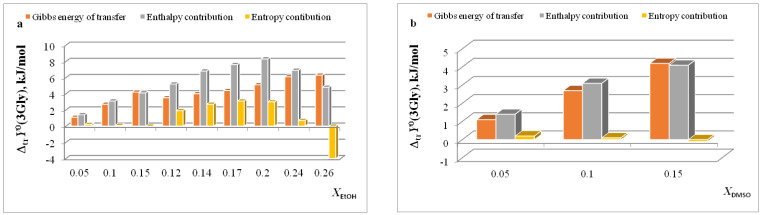
Ratios of thermodynamic parameters of transfer of 3Gly in H_2_O-EtOH (**a**) and in H_2_O–DMSO (**b**) mixture.

The diagrams in Figure 6a and Figure 7a show that the entropy and enthalpy terms almost completely compensate each other in the change in the Gibbs energy of solvation of 18C6 in aqueous organic solvents. For guest molecules, the growth of positive values of transfer enthalpies (Figure 6b, Figure 7b and Figure 8, Figure 9 and Figure 10) is mainly ahead of the change rate of *T*Δ_tr_*S*^0^. As a result, the change in the Gibbs energy of the solvation of amino acids and peptides prevails over Δ_tr_*G*^0^ (18C6).

Thus, despite the numerical advantages of the absolute values of *T*Δ_tr_*S*^0^ (18C6) and Δ_tr_*H*^0^ (18C6) over the corresponding parameters of the solvation of “guests”, the weakening of the solvation of AA and peptides dominates the weakening of the 18C6 solvation and determines the growth of stability of their molecular complexes. In turn, a significant advantage in the energy of macrocycle solvation over the change in the enthalpy of AA solvation causes an increase in the exothermicity of the complexation.

### 2.4. Thermodynamic Enthalpy–Entropy Compensation Effect in the Complexation of Cyclodextrins and 18-Crown-6 with Amino Acids in Water

**Figure 11:**
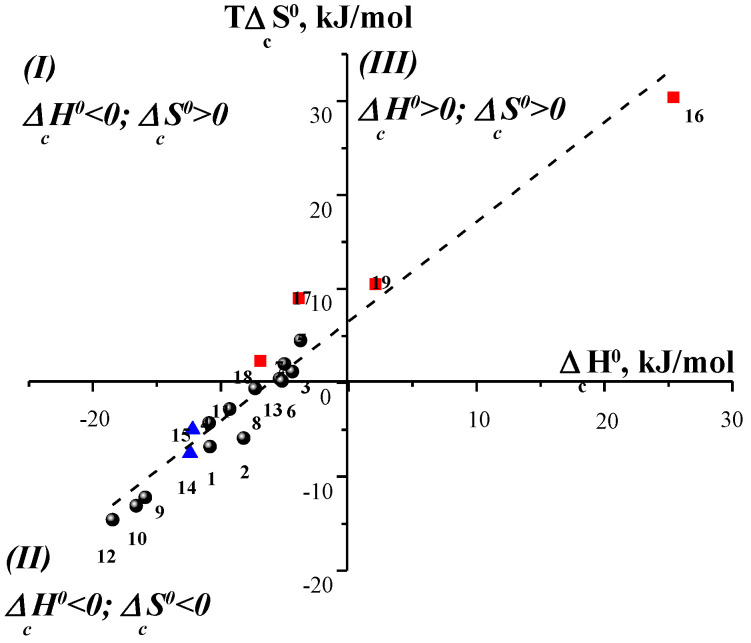
Thermodynamic enthalpy-entropy compensation effect for complex formation of amino acids with 18C6 (●), α-CD (▲) and β-CD (■) in water at 298.15 K (1 [Gly⊂18C6], 2 [Ala⊂18C6], 3 [Val⊂18C6], 4 [Leu⊂18C6], 5 [Ile⊂18C6], 6 [Pro⊂18C6], 7 [Met⊂18C6], 8 [Ser⊂18C6], 9 [Asn⊂18C6], 10 [Gln⊂18C6], 11 [Thr⊂18C6], 12 [Phe⊂18C6], 13 [His⊂18C6], 14 [Phe⊂α-CD], 15 [Trp⊂α-CD], 16 [Phe⊂β-CD], 17 [Tyr⊂β-CD], 18 [Trp⊂β-CD], 19 [His⊂β-CD]).

As it was mentioned above, the macrocyclic ligands under consideration have molecular cavities that differ in their polarity. This leads to the fact that the mechanism of interaction of CD and 18C6 with amino acids in water is different. If the amino group of all amino acids interacts with 18C6 due to electrostatic forces and H-bonds, then CDs form inclusion complexes only with aromatic amino acids, whereas their interactions with aliphatic and polar amino acids are weak. Figure 11 shows the thermodynamic enthalpy-entropy compensation for the complexation of 18C6, α-CD and β-CD with amino acids in water at 298.15 K.

As can be seen from Figure 11, all points fit into one dependence, but there is regularity in their location. The complexation of β-CD with aromatic amino acids (Phe, Trp, Tyr, His) and 18C6 with aliphatic amino acids (Val, Ile, Met, Pro) is characterized by the positive entropy changes, indicating an increase in the disorder of the system, which is caused by rearrangements of the solvent as a result of dehydration of the interacting particles and hydrophobic effects. On the contrary, complexes of α-CD with Phe and Trp as well as the complexes of 18C6 with polar amino acids belong to the second group, which is characterized by the negative changes of the enthalpy and entropy of complex formation due to the intermolecular hydrogen bonding between the polar groups of the guest and the host. Thus, from the location of the points on the dependence *T*Δ_c_*S* = *f* (Δ_c_*H*), it is possible to make an assumption about the main driving forces of complex formation and the structure of the resulting supramolecular complexes.

## 3. Conclusions

Thus, the proposed interpretation of the enthalpy-entropy compensation in the complexation of 18-crown-6 and cyclodextrins with amino acids shows that the compensation effect is not an artifact, all the obtained parameters of linear *T*Δ_c_*S*^0^ = *f*(Δ_c_*H*^0^) correlation are logical and correspond to the binding mode, proposed on the basis of a detailed study of the processes under consideration using a variety of methods.

The reactions of the formation of molecular complexes of Gly, Ala, and Phe with 18C6 in aqueous organic solvents studied by us are characterized by an increase in the positive values of the thermodynamic parameters of the transfer of the initial reagents and their molecular complexes (Δ_tr_*G*^0^ and Δ_tr_*H*^0^). The close values of Δ_tr_*G*^0^ of the complexes and 18C6 indicate the decisive role of the solvate shell of the macrocycle in the formation of the solvate shell of the molecular complexes. The differences are manifested in the determining role of the solvation contribution of the “guest” molecule in changing the stability of the complex, while the energy of molecular binding is controlled mainly by a change in the enthalpy of macrocycle solvation. The type of dependences of changes in enthalpy characteristics of solvation of the reagents is similar to the corresponding dependences of entropy solvation contributions. The largest structural and energetic changes during the transfer from water to water-organic mixtures are associated with a weakening of the solvation of the “host”, the smallest-the “guest”. It can be assumed that such ratio of entropy characteristics of the reagents is due to the conformational mobility of such relatively large cyclic molecule as 18C6. When 18C6 forms the complex, its structure apparently becomes more rigid. In comparison with crown esters, the molecules of amino acids and small peptides are less conformationally mobile.

## Data Availability

Not applicable.

## References

[1] Lehn J.-M. (2005). Design of organic complexing agents strategies towards properties. Alkali Met. Complexes Org. Ligands.

[2] Lehn J.M. (1978). Cryptates: Inclusion complexes of macropolycyclic receptor molecules. Pure Appl. Chem..

[3] The Nobel Prize in Chemistry. https://www.nobelprize.org/nobel_prizes/chemistry/laureates/1987.

[4] Pedersen C.J., Frensdorff H.K. (1972). Macrocyclic polyethers and their complexes. Angew. Chem. Int. Ed..

[5] Golcs Á., Vezse P., Ádám B.Á., Huszthy P., Tóth T. (2021). Comparison in practical applications of crown ether sensor molecules containing an acridone or an acridine unit—A study on protonation and complex formation. J. Incl. Phenom. Macrocycl. Chem..

[6] Nejad J.R., Piri A., Mohebi A., Salahshour M. (2018). Thermodynamic study of reaction of formation of Cd^2+^ cation complex and 18-crown-6 ligand in two-component mixtures of methanol/ethyl acetate, acetonitrile/ethyl acetate, tetrahydrofuran/acetonitrile, methanol/dimethyl formamide, acetonitrile/methanol and dimethyl sulfoxide/acetonitrile using conductometry method. J. Glob. Pharma Technol..

[7] Szaruga K., Fuz M., Wszelaka-Rylik M., Gierycz P. (2021). Thermodynamics of antibiotics: Natural cyclodextrin inclusion complex formation and covering of nano-metric calcite with these substances. J. Therm. Anal. Calorim..

[8] Matencio A., Caldera F., Pedrazzo A.R., Monfared Y.K., Dhakar N.K., Trotta F. (2021). A physicochemical, thermodynamical, structural and computational evaluation of kynurenic acid/cyclodextrin complexes. Food Chem..

[9] Jansook P., Ogawa N., Loftsson T. (2018). Cyclodextrins: Structure, physicochemical properties and pharmaceutical applications. Int. J. Pharm..

[10] Narayanan G., Shen J., Matai I., Sachdev A., Boy R., Tonelli A.E. (2021). Cyclodextrin-based nanostructures. Prog. Mater. Sci..

[11] Jicsinszky L., Martina K., Cravotto G. (2021). Cyclodextrins in the antiviral therapy. J. Drug Deliv. Sci. Technol..

[12] Gandhi S., Shende P. (2021). Cyclodextrins-modified metallic nanoparticles for effective cancer therapy. J. Control. Release.

[13] Jambhekar S.S., Breen P. (2016). Cyclodextrins in pharmaceutical formulations II: Solubilization, binding constant, and complexation efficiency. Drug Discov. Today.

[14] Loftsson T., Hreinsdóttir D., Másson M. (2005). Evaluation of cyclodextrin solubilization of drugs. Int. J. Pharm..

[15] Usacheva T., Sharnin V.A. (2015). A thermodynamic study of reactions of amino acids with crown ethers in nonaqueous media as examples of guest—host molecular complex formation. Russ. Chem. Bull..

[16] Usacheva T.R., Sharnin V.A. (2014). Formation of molecular complexes between 18-crown-6 and amino acids in aqueous-organic media. Russ. J. Gen. Chem..

[17] Usacheva T.R., Sharnin V.A. (2014). Effect of solvation on the complexation of 18-crown-6 with amino acids in aqueous-organic media. Russ. J. Gen. Chem..

[18] Usacheva T., Sharnin V., Chernov I., Matteoli E., Terekhova I., Kumeev R. (2012). The influence of water–ethanol mixture on the thermodynamics of complex formation between 18-crown-6 ether and l-phenylalanine. Chem. Phys. Lett..

[19] Usacheva T.R., Sharnin V.A., Chernov I.V., Matteoli E. (2013). Calorimetric investigation of the reaction of molecular complex formation of 18-crown-6 with D,L-alanine in water-ethanol mixtures. J. Therm. Anal. Calorim..

[20] Matteoli E., Lepori L., Usacheva T.R., Sharnin V.A. (2009). Thermodynamics of complex formation in mixed solvents *K* and Δ*H* for the formation reaction of [Gly18C6] at 298.15 K. J. Therm. Anal. Calorim..

[21] Usacheva T.R., Lan F.T., Sharnin V.A., Baranski A. (2013). Molecular complexation of some amino acids and triglycine with 18-crown-6 ether in H_2_O-EtOH solvents at 298.15 K. Russ. J. Inorg. Chem..

[22] Usacheva T.R., Sharnin V.A., Chernov I.V. (2013). Dependence of the thermodynamic characteristics of the complexation of alanine-18-crown-6 on the composition of water-ethanol solvent. Russ. J. Phys. Chem. A.

[23] Terekhova I.V., Lapshev P.V., Kulikov O.V. (2000). A thermodynamic analysis of selective interaction of α- and β-cyclodextrins with aromatic amino acids in water. Russ. J. Phys. Chem. A..

[24] Kulikov O.V., Terekhova I.V. (1998). Thermodynamics of complexation of α-amino acids and peptides containing nonpolar side groups with 18-crown-6 in water. Russ. J. Coord. Chem..

[25] Terekhova I.V., Kulikov O.V., Kumeev R.S., Nikiforov M.Y., Al’per G.A. (2005). ^1^H NMR Study of complexation of α- and β-cyclodextrins with some biologically active acids. Russ. J. Coord. Chem..

[26] Usacheva T.R., Ledenkov S.F., Sharnin V.A. (2002). Studies of the complex formation of silver (I) ion with 18–crown–6 in H_2_O–DMSO mixtures by calorimetric technique. J. Therm. Anal. Cal..

[27] Usacheva T., Ledenkov S., Sharnin V. (2002). Complex formation of Ag+ with polyether 18-crown-6. J. Therm. Anal. Calorim..

[28] Usacheva T.R., Ledenkov S.F., Sharnin V.A., Gzejdzak A. (2000). Thermodynamics of complexation of Ag^+^ with 18-crown-6 in water-dimethyl sulfoxide and in water-ethanol mixtures. Chem. Chem. Tech..

[29] Usacheva T.R., Kuz’Mina I.A., Sharnin V.A., Chernov I.V., Matteoli E. (2012). Thermodynamic characteristics of alanine-18-crown-6 ether complexes in binary water-acetone solvents. Russ. J. Phys. Chem. A.

[30] Usacheva T.R., Kuz’Mina I.A., Sharnin V.A., Sidorenko N.S., Voronina S.I. (2011). The influence of solvation on the formation of Ag^+^ complexes with 18-crown-6 ether in water-dimethyl sulfoxide solvents. Russ. J. Phys. Chem. A.

[31] Usacheva T.R., Kuzmina I.A., Dzumasheva N.S., Sidorenko V.A., Sharnin V.A. (2010). Solvation thermodynamics of 18-crown-6 ether in water—Ethanol binary mixture. Chem. Chem. Tech..

[32] Smirnov V.I., Badelin V.G. (2008). Enthalpies of solution of glycylglycine and diglycylglycine in aqueous alcohols at 298.15K. Thermochim. Acta.

[33] Badelin V.G., Tyunina E.Y. (2011). Effect of organic co-solvents on the solvation enthalpies of amino acids and dipeptides in mixed aqueous solutions. Russ. J. Phys. Chem. A..

[34] Smirnov V.I., Mezhevoi I.N., Badelin V.G. (2004). The enthalpies of solution of DL-α-alanine in water-alcohol mixtures at 298.15 K. Russ. J. Phys. Chem. A..

[35] Smirnov V.I., Badelin V.G. (2004). Thermochemistry of dissolving glycine, glycyl-glycine, and diglycyl-glycine in a mixed water-dimethylsulfoxide solvent at 298.15 K. Biofizika.

[36] Badelin V.G., Smirnov V.I. (2011). Influence of the composition of aqueous-alcohol solvents on the thermodynamic characteristics of l-phenylalanine dissolution at 298.15K. Thermochim. Acta.

[37] Gesse Z.F., Isaeva V.A., Sharnin V.A. (2010). The gibbs energies of transfer of glycine and glycinate ion from water into water-dimethyl sulfoxide mixtures. Russ. J. Phys. Chem. A.

[38] Remizov A., Skochilov R. (2012). Compensation and cooperative effects in H-bond thermodynamics of hydroperoxides. J. Mol. Struct..

[39] Liu Y., Han B.-H., Li B., Zhang Y.-M., Zhao P., Chen Y.-T., Wada A.T., Inoue Y. (1998). Molecular recognition study on supramolecular system. 14.1 Synthesis of modified cyclodextrins and their inclusion complexation thermodynamics with l-tryptophan and some naphthalene derivatives. J. Org. Chem..

[40] Olsson T.S.G., Ladbury J.E., Pitt W.R., Williams M.A. (2011). Extent of enthalpy-entropy compensation in protein-ligand interactions. Protein Sci..

[41] Borea P.A., Dalpiaz A., Varani K., Gilli P., Gilli G. (2000). Can thermodynamic measurements of receptor binding yield information on drug affinity and efficacy?. Biochem. Pharmacol..

[42] Starikov E.B., Nordén B. (2007). Enthalpy−entropy compensation: A phantom or something useful?. J. Phys. Chem. B.

[43] Ford D.M. (2005). Enthalpy-entropy compensation is not a general feature of weak association. J. Am. Chem. Soc..

[44] Starikov E., Nordén B. (2012). Entropy–enthalpy compensation as a fundamental concept and analysis tool for systematical experimental data. Chem. Phys. Lett..

[45] Meloun M., Ferenćíková Z. (2012). Enthalpy-entropy compensation for some drugs dissociation in aqueous solutions. Fluid Phase Equilibr..

[46] Ferrante A., Gorski J. (2012). Enthalpy–entropy compensation and cooperativity as thermodynamic epiphenomena of structural flexibility in ligand–receptor interactions. J. Mol. Biol..

[47] Exner O. (2000). Entropy–enthalpy compensation and anticompensation: Solvation and ligand binding. Chem. Commun..

[48] Rekharsky M.V., Inoue Y. (1998). Complexation thermodynamics of cyclodextrins. Chem. Rev..

[49] Gilli P., Ferretti V., Gilli G., Borea P.A. (1994). Enthalpy-entropy compensation in drug-receptor binding. J. Phys. Chem..

[50] Guo Q.-X., Zheng X.-Q., Ruan X.-Q., Luo S.-H., Liu Y.-C. (1996). Substituent effect and enthalpy-entropy compensation on the inclusion of β-cyclodextrin with 1-substituted naphthalenes. J. Incl. Phenom. Macrocycl. Chem..

[51] Inoue Y., Hakushi T. (1985). Enthalpy–entropy compensation in complexation of cations with crown ethers and related ligands. J. Chem. Soc. Perkin Trans. 2.

[52] Al Omari M.M., Zughul M.B., Davies J.E.D., Badwan A.A. (2007). Thermodynamic enthalpy–entropy compensation effects observed in the complexation of basic drug substrates with β-cyclodextrin. J. Incl. Phenom. Macrocycl. Chem..

[53] Rekharsky M.V., Inoue Y. (2002). Solvent and guest isotope effects on complexation thermodynamics of α-, β- and 6-amino-6-deoxy-β-cyclodextrins. J. Am. Chem. Soc..

[54] Meo P.L., D’Anna F., Gruttadauria M., Riela S., Noto R. (2004). Thermodynamics of binding between α- and β-cyclodextrins and some p-nitro-aniline derivatives: Reconsidering the enthalpy–entropy compensation effect. Tetrahedron.

[55] Leung D.H., Bergman R.G., Raymond K.N. (2008). Enthalpy−entropy compensation reveals solvent reorganization as a driving force for supramolecular encapsulation in water. J. Am. Chem. Soc..

[56] Grunwald E., Steel C. (1995). Solvent reorganization and thermodynamic enthalpy-entropy compensation. J. Am. Chem. Soc..

[57] Usacheva T.R., Sharnin V.A., Matteoli E., Taylor J.C. (2014). Influence of water-dimethyl sulfoxide medium on complex-forming properties of crown ether 18-crown-6. Advances in Chemistry Research.

[58] Shormanov V.A., Sharnin V.A., Kutepov A.M. (1998). Achievements and Problems of Solvation Theory: Structural and Thermodynamic Aspects.

[59] Sharnin V.A., Usacheva T.R., Kuzmina I.A., Gamow G.A., Alexandriiskii V.V. (2019). Complexation in Non-Aqueous Media: A Solvation Approach to Describing the Role of a Solvent.

[60] Krestov G.A. (1994). Ionic Solvation.

[61] Usacheva T.R., Chernov I.V., Sharnin V.A., Voronina S.I., Matteoli E. (2012). Molecular complex formation between l-phenylalanine and 18-crown-6 in H2O–DMSO solvents studied by titration calorimetry at T = 298.15 K. J. Therm. Anal. Calorim..

[62] Usacheva T.R., Kuzmina I.A., Sharnin V.A., Chernov I.V., Matteoli E. (2012). Influence of the composition of aqueous dimethylsulfoxide solvent on thermodynamics of complexing between 18-crown-6-ether and D,L-alanine. Russ. J. Phys. Chem. A.

[63] Usacheva T.R., Kuz’Mina I.A., Sharnin V.A., Chernov I.V., Matteoli E. (2011). The influence of the composition of an aqueous-acetone solvent on the thermodynamic characteristics of complex formation of 18-crown-6 ether with glycine. Russ. J. Phys. Chem. A.

[64] Usacheva T.R., Sharnin V.A., Matteoli E., Vojak W. (2013). Molecular complexation of clycine by 18-crown-6 in aqueous-organic solvent: A solvation-thermodynamic study. Glycine: Biosynthesis, Physiological Functions and Commercial Uses.

[65] Kustov A.V., Batov D.V., Usacheva T.R., Sharnin V.A. (2016). Calorimetry of Non-Electrolyte Solutions: Theoretical Foundations, Experiment, Data Analysis.

